# SHetA2 Attack on Mortalin and Colleagues in Cancer Therapy and Prevention

**DOI:** 10.3389/fcell.2022.848682

**Published:** 2022-02-23

**Authors:** Doris Mangiaracina Benbrook

**Affiliations:** Stephenson Cancer Center, Obstetrics and Gynecology Department, Gynecologic Oncology Section, University of Oklahoma Health Sciences Center, Oklahoma City, OK, United States

**Keywords:** mortalin, Hsp70, hsc70, Grp78, cancer, mitochondria, metabolism, apoptosis

## Abstract

Heat Shock Proteins of the 70-kDa family (HSP70s) do not cause cancer by themselves, but instead protect cells as they transform into cancer. These molecular chaperones bind numerous client proteins and utilize ATP hydrolysis to facilitate proper protein folding, formation of functional complexes and cellular localizations, or degradation of irreparably damaged proteins. Their transient upregulation by stressful situations avoids induction of programmed cell death. Continued upregulation of the mortalin, heat shock cognate (hsc70) and glucose regulated protein 78 (Grp78) support cancer development and progression by supporting pro-proliferative and metabolic functions and repressing pro-death functions of oncoproteins and tumor suppressor proteins. This review describes the discovery and development of a lead anti-cancer compound, sulfur heteroarotinoid A2 (SHetA2, NSC726189), which was originally developed to bind retinoic acid receptors, but was subsequently found to work independently of these receptors. The discovery and validation of mortalin, hsc70 and Grp78 as SHetA2 target proteins is summarized. The documented and hypothesized roles of these HSP70 proteins and their clients in the mechanism of SHetA2 inhibition of cancer without toxicity are discussed. Use of this mechanistic data to evaluate drug action in a cancer clinical trial and develop synergistic drug combinations is explained. Knowledge needed to optimize SHetA2 analogs for use in cancer therapy and prevention is proposed as future directions.

## Introduction

Cancer cells would face extreme difficulty evolving and surviving the hypoxic- and nutrient-deprived conditions in tumor microenvironments were it not for mortalin and other family members of the 70-kDa heat shock protein A family (HSP70s) (Vostakolaei et al., 2019; Rai et al., 2021). Under healthy conditions, HSP70s facilitate folding of newly-synthesized proteins, refolding of misfolded proteins, protein intracellular localization, functional protein complex formation and stability, and protein degradation. HSP70s are transiently induced by a wide variety of stresses until homeostasis can be restored (Kodiha et al., 2005; Liu et al., 2012). Cancer cells that develop constitutive elevation of HSP70s have a survival advantage that facilitates tumorigenesis, metastases and resistance to radiation and chemotherapy. Thus, the HSP70s are rational targets for development of cancer prevention and treatment drugs. In particular, mortalin represents a promising drug target candidate based on the selective expression of mortalin/client protein complexes in cancer cells and the minimal-to-no toxicity of mortalin-inhibitory drugs on healthy cells and in animal models (Deocaris et al., 2009; Lu et al., 2011; Kabirov et al., 2012; Garg et al., 2020; Sari et al., 2021). Toxicity of the MKT-077 compound in clinical trials appears to have been caused by non-specific off target effects (Garg et al., 2020). This review describes the discovery and development of a small molecule investigational new drug, sulfur heteroarotinoid A2 (SHetA2, pronounces “Ess-Het-Aye-Two), which inhibits cancer development and growth through disruption of complexes formed between mortalin and other HSP70 proteins with their client proteins.

## Flexible-Heteroarotinoid Discovery

**FIGURE 1:**
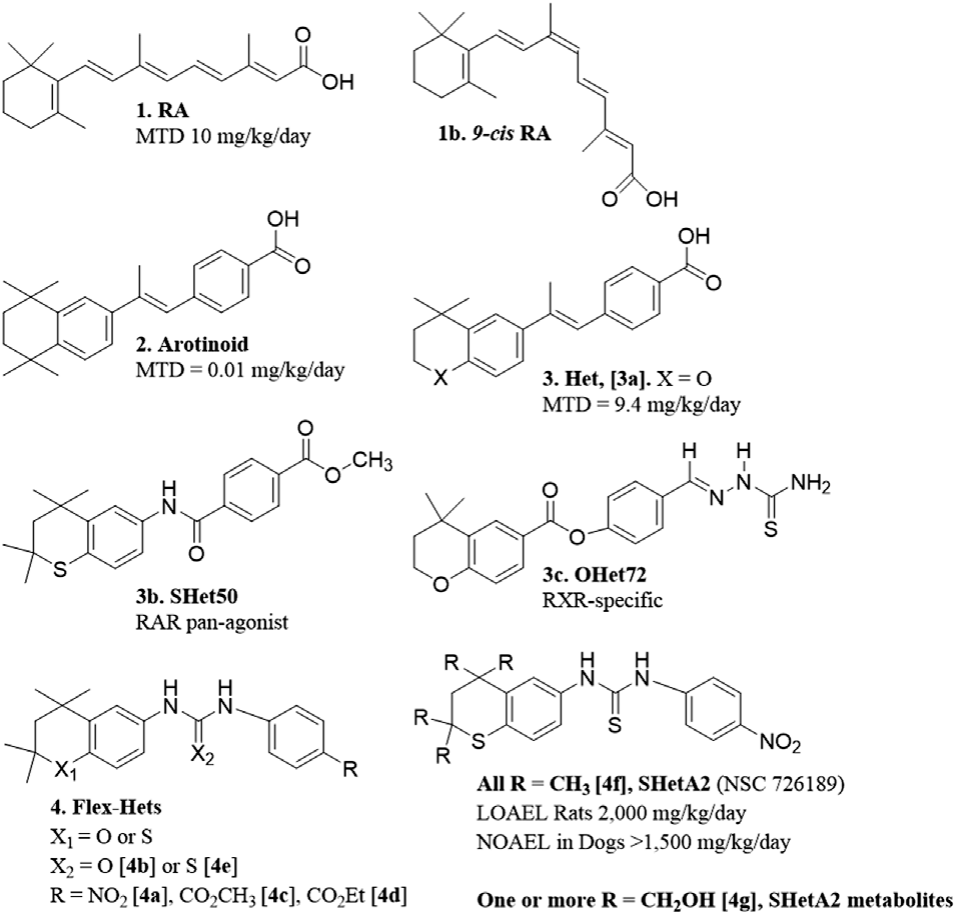
Structures and toxicity endpoints of hets and flex-hets.

The major objective in the drug discovery program that produced SHetA2 was to develop anti-cancer drugs with little-to-no toxicity. Originally, this program was focused on designing retinoids, which are synthetic versions of retinoic acid (RA) (Figure 1 [**1**]). Although RA is effective in treating some forms of cancer, clinical use of RA is limited by toxicity and development of resistance (Connolly et al., 2013). Retinoid toxicities in clinical trials involve weight gain and unexplained fever associated with elevated white blood cell counts, difficulty breathing associated with interstitial pulmonary infiltrates and effusion in the pleural and pericardial cavities, episodic hypotension and acute renal failure, headache and bone pain (Larson and Tallman, 2003). It was hypothesized that the high flexibility of the RA tetraene side chain allowed multiple conformations with different activities, which included causes of the toxicity (Dawson et al., 1985). Thus, an early retinoid design was to restrict the flexibility of the RA tetraene side chain by incorporating it into an aromatic ring. These aromatic compounds, called arotinoids (Figure 1 [**2**]), exhibited potent RA biological activity, but 1000-fold higher toxicity in comparison to RA (Lindamood et al., 1990). Preclinical evidence suggested that the greater toxicity of an arotinoid is due to its slower disappearance compared to RA (Pignatello et al., 1999). Then, the first generation of heteroarotinoids (Hets) (Figure 1 [**3**]) were designed to produce arotinoid analogs with reduced toxicity while maintaining the beneficial biological activities. Based on the assumption that oxidized metabolites of the arotinoids contributed to the toxicity, the strategy was to substitute the *gem*-dimethyl group of the arotinoid aromatic ring with an oxygen or sulfur heteroatom to prevent oxidation at this site of the molecule. This strategy was successful and produced Hets that were 1000-fold less toxic than arotinoids and equivalent in toxicity to RA (Figure 1 [**3a**]) (Benbrook et al., 1997b). In this toxicity study, the arotinoid [**2**] caused dose-dependent classical retinoic acid toxicities including bone fractures, alopecia, elevated white blood cell counts and triglycerides, reduced organ-to-body weight ratios for testes, increased organ-to-body weight ratios for thymus, spleen and adrenal gland, and enlarged lymph nodes. These toxicities were also observed for the Het [**3b**], but only at higher doses, longer treatment times and in fewer mice, leading to the 1000-fold higher maximally-tolerated dose (MTD) in comparison to the arotinoid [**2**].

Based on this promising result, the next effort was to design Hets with reduced toxicity. At this point, it was well established that RA and synthetic retinoids exerted their biological effects by regulating the functions of nuclear receptors that act as transcription factors. There are three nuclear retinoic acid receptors (RAR*α*, RAR*β* and RAR*γ*) and three retinoid X receptors (RXR*α*, RXR*β* and RXR*γ*), which function as dimers to regulate transcription of genes containing retinoic acid response elements (RAREs) (Benbrook et al., 2014). The degree of RARE transactivation induced by retinoids in cancer cells was shown to directly correlate with the extent of growth inhibition caused by retinoids in organotypic 3-dimensional (3-D) cancer cultures (Benbrook et al., 1997a). It was then hypothesized that individual retinoic acid receptor subsets mediate the anti-cancer efficacy, while other subsets are responsible for the toxicities. Receptor selective Het structures were designed to modify the rigid structure to fit selectively into individual RARs or RXRs by including various substitutions around basic Het backbone (Benbrook et al., 1998). These efforts produced receptor-selective Hets with reasonable anti-cancer activity and acceptable toxicity (Dhar et al., 1999; Zacheis et al., 1999). In a head and neck squamous cell carcinoma (HNSCC) cell line xenograft model at a dose of 10 mg/kg/day, an RAR pan-agonist Het called SHet50 [**3b**] had greater growth inhibition activity in comparison to an RXR-specific Het called OHet72 [**3c**] and RA [**1**] (Zacheis et al., 1999). These Hets were not further developed because a more potent second-generation class of Hets with greater anti-cancer activity without elevated toxicity was discovered and chosen for further development as described below.

The strategy for the next generation Hets diverged from the original by incorporating a urea or thiourea linker to increase the compound flexibility, and also screening the compounds with a cell-based, instead of an RAR/RXR activation-based, assay. In ovarian cancer organotypic cultures produced from established cell lines and primary cultures, these flexible heteroarotinoids (Flex-Hets) (Figure 1 [**4**]) inhibited proliferation and induced apoptotic cell death, while the effects of the first generation more-rigid Hets were limited to growth inhibition (Guruswamy et al., 2001). This difference was associated with lack of RAR/RXR activation by Flex-Hets, suggesting that the Flex-Hets had a novel mechanism of action (Guruswamy et al., 2001). The use of 3-D organotypic cultures allowed modeling of Flex-Het tissue level effects, which revealed drug-induced formation of more-orderly tissue structures including mucin-producing glands in ovarian cancer cultures and tubules in kidney cancer cultures in association with upregulation of Mucin-1 and E-Cadherin, respectively (Guruswamy et al., 2001; Liu et al., 2009). Animal studies validated that Flex-Het inhibition of growth and induction of differentiation in association with upregulation of MUC-1 and E-Cadherin, and apoptosis also occurs in xenograft tumors generated from ovarian and kidney cancer cell lines (Benbrook et al., 2005; Liu et al., 2009). Flex-Hets did not induce toxicity, mutagenicity, teratogenicity, skin irritation or liver damage in animal studies, which is consistent with the differential effects of Flex-Hets on cancer over healthy cells and the RAR/RXR-independence of these compounds (Mic et al., 2003; Benbrook et al., 2005; Liu et al., 2009; Doppalapudi et al., 2012).

## Flex-Het Differential Effects on Cancer Over Healthy Cells Involve Mitochondria

The differential level of cytotoxicity caused by Flex-Hets on cancer over healthy cells was observed for multiple organ types. In the ovarian studies, cultures generated from a benign ovarian cyst, healthy endometrial cells or oral non-cancerous fibroblasts and immortalized ovarian surface epithelial cells were less sensitive to Flex-Het growth inhibition and induction of cell death when compared to ovarian cancer cell lines and a primary culture generated from a borderline/low malignant potential ovarian tumor (Liu et al., 2007; Liu et al., 2009). Upregulation of cell surface phosphatidyl serine on the plasma membrane with moderate cell uptake of propidium iodide (PI) into the cell documented that the mechanism of SHetA2-induced cancer cell death involved apoptosis (Chun et al., 2003; Liu et al., 2007). The Flex-Het sensitivities of two ovarian cancer cell lines were associated with generation of reactive oxygen species (ROS) and cell death (Liu et al., 2004). Subsequent mechanistic studies revealed that the differential cytotoxicities of Flex-Hets on cancer compared to healthy cells involved rapid mitochondrial swelling and reduction of mitochondrial membrane potential in ovarian cancer, but not healthy cells (Liu et al., 2007). These effects were associated with reduction of anti-apoptotic (Bcl-2 and Bcl-xl) and no effect on pro-apoptotic (Bax) Bcl-2 protein family members in cancer cells. In contrast, healthy cells exhibited no change in Bcl-xl or Bax levels and increased Bcl-2 levels after 16 and 24 h of Flex-Het treatment. Use of two anti-oxidants with different mechanisms of quenching ROS, butylated hydroxyanisol and Mn (III) tetrakis (4-benzoic acid) porphyrin, prevented ROS generation in the cytosol and mitochondria, respectively, but did not reduce apoptosis caused by Flex-Het treatment. These results indicated that ROS were not a cause of the apoptosis, and instead only a consequence of the mitochondrial damage caused by Flex-Hets. Furthermore, protein synthesis was not required for Flex-Het induction of mitochondrial damage or apoptosis, indicating that mitochondrial components are direct targets of the drugs.

A similar mechanism of differential induction of cell death in cancer compared to healthy cells was observed in kidney epithelial cells (Liu et al., 2009). Flex-Het induction in kidney cancer cell lines, but not in the non-cancerous HK-2 cell line or primary cultures of epithelial cells derived from normal kidney, was associated with cleavage of caspase three and (PARP-1) and reduction of Bcl-2, but not Bax. Structure activity relationship studies, identified that Flex-Hets with NO_2_ group substitutions on the single aromatic ring [**4a**] and urea linkers [**4b**] exhibited the more potent and effective cytotoxity compared to Flex-Hets with carboxymethyl [**4c**] or carboxyethyl [**4d**] substitutions and thiourea linkers [**4e**], respectively (Figure 1). Furthermore, decreasing the polarity of the compounds to increase bioavailability did not prevent the differential toxicity of Flex-Hets against cancer compared to healthy cells.

## Choice of SHetA2 as the Lead Flex-Het Compound

The SHetA2 analog (Figure 1 [**4f**]) was chosen as the lead Flex-Het compound for further study because it was the most potent Flex-Het for cytotoxicity against cancer cells, while retaining a much-reduced effect on healthy cells (Chun et al., 2003; Liu et al., 2004). Example comparisons of SHetA2 efficacies and toxicities with other retinoids and Hets are summarized in Table 1. SHetA2 also exhibited greater inhibition of ovarian cancer cell line xenograft growth in comparison to a similar Flex-Het called SHetA4, which differs from SHetA2 by a single substitution of a carboxyethyl group in place of the NO_2_ group (Benbrook et al., 2005). This greater tumor growth inhibition activity of SHetA2 in comparison to SHetA4 was associated with higher induction of gland formation, MUC-1 expression and apoptosis in the tumors. Consistent with the cell culture data, neither SHetA2 nor SHetA4 caused elevation of serum alanine aminotransferase (ALT) as a biomarker of liver toxicity. Screening of multiple cell lines demonstrated SHetA2 dose-responsive inhibition of growth in the low micromolar concentration range for cervical cancer cell lines, HNSCC, and all cancer types (Leukemia, central nervous system cancer, kidney cancer, non-small cell lung cancer, Melanoma, prostate cancer, colon cancer, ovarian cancer and breast cancer) included in the National Cancer Institute (NCI) 60-cell line panel (Chun et al., 2003; Benbrook et al., 2005). Independence of SHetA2 from RAR/RXR activation in these studies was confirmed by the inability of an RAR antagonist or an RXR antagonist to alter SHetA2-induced apoptosis and the lack of RAR/RXR upregulation in SHetA2-treated cells.

**TABLE 1:** Comparison of SHetA2 activities and toxicities with retinoids, Hets and other Flex-Hets.

Compound	Effective *in vitro* dosing			Effective *in vivo* dosing	Toxicity
	Cervical cancer	Kidney cancer	Normal kidney epithelial cells		
RA [**1**]	ND	4.6–23.6 µM IC_50_ 0–7% Efficacy	6 µM IC_50_ 16% Efficacy	10 mg/kg/day^A^	10 mg/kg/day in 30 day mouse study
9-cis RA [**1b**]	5.5–8.6 µM IC_50_ 24–47% Efficacy	ND	ND	ND	ND
Arotinoid [**2**]	ND	ND	ND	ND	0.001 mg/kg/day MTD in 30 day mouse study
Het [**3a**]	ND	ND	ND	ND	9.4 mg/kg/day MTD in 30 day mouse study
SHet50 [**3b**]	3.9–11.9 µM IC_50_ 15–67% Efficacy	ND	ND	10 mg/kg/day^A^	ND
SHetA4 [**4, X**_**1**_ **= S, X**_**2**_ **= O, R = CO**_**2**_ **Et]**	4.3–6.5 µM IC_50_ 59–85% Efficacy	2.6–5.1 µM IC_50_ 45–69% Efficacy	5.2–7.6 µM IC_50_ 26–40% Efficacy	10 mg/kg/day^B^	ND
SHetA3 [**4, X**_**1**_ **= S, X**_**2**_ **= S, R = CO**_**2**_ **Et**]	4.8–7.5 µM IC_50_ 42–84% Efficacy	7.2–7.3 µM IC_50_ 54–57% Efficacy	5.1–5.9 µM IC_50_ 26–40% Efficacy	ND	ND
SHetA2 [**4f**]	2.3–3.9 µM IC_50_ 58–92% Efficacy	4.9–7.6 µM IC_50_ 72–84% Efficacy	4.5–4.6 µM IC_50_ 37–51% Efficacy	10 mg/kg/day^B^	>1,500 mg/kg/day NOAEL in 28 day dog study

IC_50_: Concentration that causes 50% growth inhibition of cell cultures, Efficacy: Maximal amount of growth inhibition of cell cultures. ^A^HNSCC Line Xenografts, ^B^Ovarian Cancer Cell Line Xenografts. Compounds above the dotted line are retinoids and those below the dotted line are retinoic acid receptor-independent.

## Preclinical Development of SHetA2 as the Lead Flex-Het Compound

Based on these promising results, the NCI Rapid Access to Intervention Development (RAID) Program awarded a grant to produce the preclinical pharmacology and toxicology data needed for submission of an Investigational New Drug (IND) application to the US Food and Drug Administration (FDA) to allow the first in human clinical trial of SHetA2. The first accomplishment of this effort was development of a highly sensitive high performance liquid chromatography (HPLC)/UV method for quantification of SHetA2 in plasma (Zhang et al., 2006). This method was then used to measure the oral bioavailability of SHetA2. An oral route of administration was chosen because this route was effective at reducing growth in the previously-mentioned tumor xenograft studies, and is preferred for use in clinical cancer chemoprevention approaches over more-invasive methods of drug administration. Comparison of an intravenous bolus of 20 mg/kg SHetA2 with oral gavage of SHetA2 dissolved in sesame oil at 20 and 60 mg/kg produced estimates of 15 and 19% oral bioavailability in mice, respectively. The plasma SHetA2 concentration data fit into a two-compartment deconvolution model and the overall pharmacokinetic behavior of SHetA2 was reported to be favorable for further development of this lead compound as a drug.

The RAID program also developed a liquid chromatography/tandem mass spectrometry (LC-MS/MS) assay for measurement of SHetA2 and its metabolites *in vitro* and *in vivo* (Liu et al., 2008). Using this assay, SHetA2 metabolites in rat and human liver microsomes were identified to be hydroxylated on one or more of the four SHetA2 aliphatic methyl groups (Figure 1 [**4g**]). Addition of glutathione (GSH) to the microsomes produced SHetA2 GSH-adducts. In validation of these findings, hydroxylated metabolites of SHetA2 were detected in plasma and liver, and GSH-adducts of SHetA2 were detected in liver, after intravenous administration of 40 mg/kg SHetA2 in a rat.

The NCI Rapid Access to Preventive Intervention Development (RAPID) program awarded a grant for focused development of SHetA2 as a cancer chemoprevention agent. In this project, oral toxicology and pharmacokinetic studies of SHetA2 were conducted in rats and dogs using a LC-MS/MS assay (Kabirov et al., 2012). Daily administration of SHetA2 suspended in 1% aqueous methylcellulose/0.2% tween 80 for 28 days resulted in a lowest observed adverse effect level (LOAEL) of 2,000 mg/kg/day SHetA2 in rats. At this 2,000 mg/kg/day highest dose tested, decreased food consumption and body-weight gains, decreased relative prostate weights and increased relative adrenal gland weights (in females only) were observed. No significant SHetA2 toxicities were observed on neurological, clinical chemistry, hematology, and coagulation parameters conducted at 4 weeks of treatment. For the 28 day dog toxicity study, an oral formulation of SHetA2 in 30% aqueous Solutol HS 15 (now called Kolliphor HS 15) resulted in a no observed adverse effect level (NOAEL) of >1,500 mg/kg/day. This dose was the highest evaluated and resulted in no significant changes in any of the parameters studied. In both the rat and dog studies, systemic exposure to SHetA2 was confirmed and monohydroxylated SHetA2 (Figure 1 [4g]) was identified as the major metabolite in plasma. The carboxymethylcellulose formulation used in rats afforded poor bioavailability (<1%). Because the formulation in Kolliphor HS15, which is a self-emulsifying drug delivery system (SEDDS) (Singh et al., 2020), allowed a much higher bioavailability, this formulation was utilized as the basis for all subsequent studies.

## Identification of SHetA2 Mechanism of Action

Further development of SHetA2 as a drug was hindered by poor mechanistic understanding of how it inhibits cancer. The lack of SHetA2 induction of RAR/RXRs and related toxicities, and the inability of RAR/RXR antagonists to interfere with SHetA2 activities indicated that this compound acts through a mechanism that is independent of RAR/RXRs. To identify the mechanism of SHetA2 action, magnetic microspheres were produced and used to isolate SHetA2-binding proteins from cancer cell protein extracts (Benbrook et al., 2013b). A synthetic scheme was developed and utilized to produce a hydroxylated SHetA2 metabolite (Nammalwar et al., 2011), which was further modified to facilitate its attachment to a magnetic microsphere (Benbrook et al., 2013b). A polyethylene glycol 4 (PEG4) linker was incorporated in between the SHetA2 and microsphere to avoid interference of the microsphere with SHetA2 binding to proteins. The SHetA2-conjugated magnetic microspheres or empty-magnetic microspheres were incubated with whole cell protein extracts from the A2780 ovarian cancer cell line. A magnet was used to collect the bound proteins. Excess free SHetA2 compound was used to elute the proteins from the microspheres. SDS-PAGE gels revealed a 72 kDa band in the eluent from the SHetA2-conjugated microspheres that was not present, or present at a lower intensity, in the empty-microspheres depending on the conditions. Incubation and washing conditions were optimized to minimize non-specific protein binding and maximize specific SHetA2-protein binding to the microspheres. Excision and MS analysis of the 72 kDa band identified three related heat shock proteins (HSPs) to be present in the band from the SHetA2-microsphere eluent and not in the band from the empty-microsphere eluent. These proteins, glucose-regulated protein 78 (Grp78), heat shock cognate 70 (hsc70) and mortalin encoded by the *HSPA5*, *HSPA8* and *HSPA9* genes, respectively, were also present at significantly greater levels in total eluents (not subjected to SDS Gel band isolation) from the SHetA2-microspheres compared to the empty microspheres in an independent experiment using a different MS system.

The known functions of Grp78, hsc70 and mortalin fit well into the existing mechanistic knowledge of SHetA2 like missing pieces into a jigsaw puzzle (Rai et al., 2021). These proteins belong to the HSP70 family of molecular chaperones that have homologous protein structures consisting of ATPase and peptide binding domains. They function as molecular chaperones by working in concert with other HSPs and co-factors to fold newly-synthesized proteins and re-fold misfolded or unfolded proteins. Each HSP70 has a range of cellular localizations and functions, some of which are redundant and others that are unique. Grp78 is primarily localized in the endoplasmic reticulum (ER) where it controls the unfolded protein response (UPR), a survival mechanism that rebalances the cellular folding and degradation machinery to alleviated excessive buildup of unfolded or misfolded proteins. Therefore, SHetA2 binding to, and potential inhibition of, Grp78 is a logical mediator of the ER swelling and upregulation of UPR proteins observed in SHetA2-treated cancer cells (Lin et al., 2008a; Benbrook and Long, 2012). The hsc70 protein is primarily localized in the cytoplasm and is a likely target involved in SHetA2 regulation of the cell cycle as described in detail below. Mortalin is primarily localized in the mitochondria and is a likely mediator of SHetA2-induced mitochondrial damage as described in detail below. Co-immunoprecipitation experiments, demonstrated that SHetA2 disrupts complexes of mortalin with two of its client proteins, p53 and p66shc, which suggests that SHetA2 interferes with the molecular chaperone function of its HSP70 binding proteins. Upregulation of the HSP70/client proteins in cancer compared to healthy cells represents a rational explanation for the differential toxicity of SHetA2 on cancer compared to healthy cells.

**FIGURE 2:**
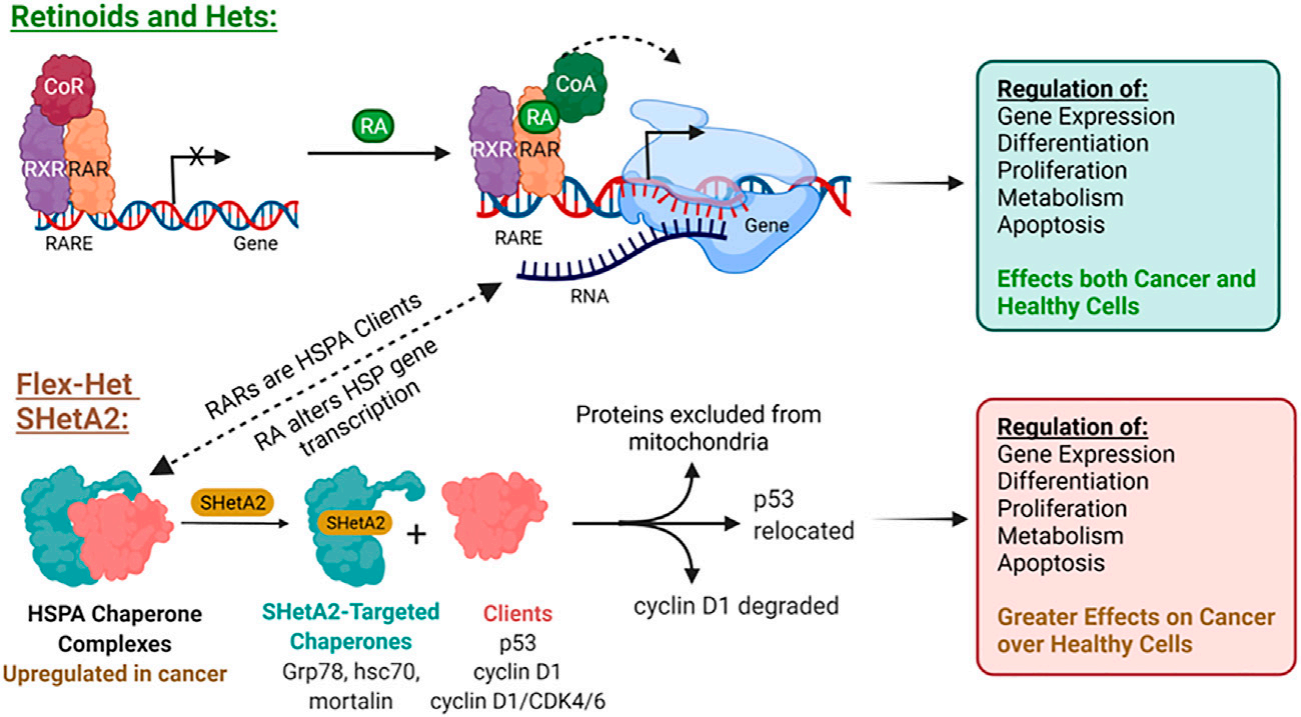
Differences in mechanisms of RA and the Flex-Het SHetA2.

This SHetA2 targeting of HSP70 proteins is different from the mechanism of retinoid biological activities (Figure 2). In contrast to SHetA2, retinoids have not been shown to bind HSP70 proteins, however RA [**1**] treatment has been shown to cause Grp78 dephosphorylation, and either increased or reduced Grp78 expression, depending on the cell type (Freyria et al., 1995; Xu et al., 2007; Hsu et al., 2008; Mandili et al., 2011). In contrast to retinoids, SHetA2 does not activate or induce expression of the RAR/RXR receptors (Guruswamy et al., 2001; Benbrook et al., 2005). It appears that HSP70 proteins support retinoid biological programs, and theoretically, SHetA2 has the potential to reduce this collaboration. Mortalin binds and protects RARs from proteasomal degradation (Shih et al., 2011). Multiple heat shock proteins and both mortalin and Grp78 appear to support retinoic acid regulation of normal and abnormal differentiation during embryogenesis based on their expression being altered during these RA [**1**]-induced processes (Zhu et al., 2012; Zhu et al., 2013). Thus, HSP70 proteins could support RA [**1**] regulation of differentiation by maintaining folding and function of RAR/RXRs and folding the newly-synthesized proteins induced by RA [**1**] treatment. Concern that SHetA2 disruption of mortalin and Grp78 complexes could potentially cause teratogenesis is reduced by the lack of SHetA2 alteration of cellular RAR/RXR levels and lack of SHetA2 teratogenesis in a mouse model (Mic et al., 2003; Benbrook et al., 2005). Furthermore, SHetA2 has been shown to reverse the abnormal differentiation phenotype and induce development of normal differentiated structures, such as glands and tubules, in organotypic cultures and xenograft tumors (Guruswamy et al., 2001; Liu et al., 2009). To further develop SHetA2 as an anti-cancer drug, this HSP70-based mechanism of action needed to be studied in more detail.

## Role of Mortalin/Client Protein Complexes in SHetA2 Differential Effects on Cancer Over Healthy Cells

Mortalin/p53 complexes were evaluated in the SHetA2 mechanism because mortalin appears to protect cells from p53-induced apoptotic cell death, while allowing sufficient p53 to drive elevation of cancer metabolism and growth. In healthy tissues, wild type p53 protein is transiently elevated by various stresses until the p53 function alleviates the inducing stress. In situations where the stress cannot be alleviated, continued high levels of p53 induce transcription of apoptosis-inducing genes, and activate Bax-formation of mitochondrial pores to release apoptosis-inducing factors (Chipuk et al., 2004; Aubrey et al., 2018). Stress-induced levels of mortalin can prevent p53-induction of apoptosis by binding p53 and sequestering it away from the nucleus (Lu et al., 2011). Because high grade serous ovarian cancer (HGSOC) appears to arise in the fallopian tube from lesions that overexpress p53 protein (Corzo et al., 2017) and 96–100% of HGSOC lesions contain p53 mutations (Cancer Genome Atlas Research Network, 2011; Vang et al., 2016), this cancer was chosen for studies of the role of mortalin/p53 complexes in the SHetA2 mechanism (Ramraj et al., 2019). Evaluation of *TP53* gene mutations in The Cancer Genome Atlas (TCGA) data of HGSOC tumors found the majority (58%) of the tumors were missense mutations associated with elevated p53 proteins levels. Immunohistochemical staining of tissue microarrays demonstrated sequential elevation of mortalin in benign to borderline to cancerous ovarian tissues. SHetA2 treatment caused nuclear and mitochondrial accumulation of p53 and apoptosis in ovarian cancer cell lines, but not in primary cultures of human fallopian tube secretory epithelial cells. Oral treatment with SHetA2 significantly reduced establishment and growth of HGSOC tumors in the peritoneum of mice in a model that mimics maintenance therapy, which is now used as a secondary chemoprevention approach in standard-of-care to prevent ovarian cancer recurrence after tumor debulking and primary therapy.

## SHetA2 Disruption of Mortalin Support of Mitochondria and Metabolism

Mortalin/client protein complexes also have been implicated in the mechanism by which SHetA2 damages mitochondria and alters metabolism in endometrial cancer cells (Chandra et al., 2021). Mortalin serves a vital role in mitochondria biogenesis and function by providing the ATPase function to the machinery that imports the 1,000 mitochondrial proteins that are encoded in the nuclear genome (Zhao and Zou, 2021). Thus, disruption of mortalin/client proteins is a logical upstream causation of the mitochondrial swelling and loss of membrane potential caused by SHetA2 and related Flex-Hets in ovarian cancer cells (Liu et al., 2007). SHetA2 also caused loss of mitochondrial membrane potential and reduction of mitochondrial fission and fusion proteins in endometrial cancer cells (Chandra et al., 2021).

SHetA2 treatment of endometrial cancer cells disrupted mortalin binding to metabolic enzymes, aldehyde dehydrogenase 18 family member A1 (ALDH18A1), cytidine triphosphate synthetase (CTPS), malate dehydrogenase (MDH1), and enoyl coenzyme A hydratase, short chain, 1, mitochondrial (ECHS1) (Chandra et al., 2021). ALDH18A1 is a mitochondrial enzyme that catalyzes the first two steps in *de novo* biosynthesis of amino acids proline, ornithine, citrulline, and arginine. Currently, this is the only report of mortalin directly binding to these specific proteins. Overexpression of ALDH18A1 in cancer supports accelerated metabolism, angiogenesis, metastases and chemotherapy resistance (Tang et al., 2018; Ding et al., 2020; Guo et al., 2020; Rohlenova et al., 2020; Shenoy et al., 2020; Kocher et al., 2021). Complexes of CTPS1 and inosine monophosphate dehydrogenase type 2 (IMPDH2), called cytoophidia or rods and rings, regulate key steps in CTP and GTP biosynthesis. These complexes are induced by glutamine deprivation and their elevated levels in kidney cancer were associated with metastases and patient survival (Calise et al., 2014; Ruan et al., 2020). Elevation of MDH1 supports the aerobic glycolysis in cancer cells by providing NAD and converting glucose to malate, and its urinary levels are associated with non-small-cell lung cancer (Hanse et al., 2017; Ma et al., 2021). ECHS1 oxidizes fatty acids (FAs) and branched-chain amino acids (BCAAs), which removes their activation of mammalian target of rapamycin (mTOR) signaling, a well-established factor that drives increased protein synthesis and mitochondrial biogenesis in cancer. Thus, it is not surprising that, in contrast to the metabolic enzymes described above, ECHS1is inhibited by overnutrition and its decrease in cancer supports enhanced metabolism (Zhang et al., 2017). Overall, inhibition of mortalin interaction with these metabolic enzymes has the potential to disrupt their support of the elevated protein and DNA synthesis and glycolysis in cancer cells. Consistent with the disruptions of mortalin binding to metabolic enzyme client proteins, SHetA2 treatment forced endometrial cancers cells into a quiescent state by inhibiting both oxidative phosphorylation and glycolysis (Chandra et al., 2021). Identification and bioinformatic analysis of proteins altered by this SHetA2 treatment indicated that down-stream targets of Grp78 and hsc70 involved in glycolysis, gluconeogenesis, eukaryotic initiation factor 2 (EIF2) and ER stress were also significantly affected by SHetA2 treatment of endometrial cancer cells (Chandra et al., 2021).

SHetA2 also disrupted mortalin binding to the inositol trisphosphate receptor (IP3R) in endometrial cancer cells (Chandra et al., 2021). The interaction of mortalin with IP3R facilitates calcium (CA^2+^) transfer from the ER to the mitochondria by forming a bridge between IP3R on the ER membrane and the voltage-dependent anion channel (VDAC) on the outer mitochondrial membrane (Szabadkai et al., 2006). CA^2+^ is a vital cell signaling molecule whose binding proteins in the mitochondrial control cell fate through regulation of cell death and metabolism. Disruption of CA^2+^ cell signaling and homeostasis is a characteristic of cancers that drives uncontrolled cell growth and avoidance of consequent cell death programs (Danese et al., 2021).

## Mechanisms of SHetA2-Induced Cancer Cell Death

There exist multiple natural programmed mechanisms through which cell death can occur (Galluzzi et al., 2018). The intrinsic apoptosis mechanism is initiated at damaged mitochondria. Factors which can cause cell death are readily available in mitochondria without the need for new RNA or protein synthesis. While inside the mitochondria, these factors serve vital roles in metabolism, however upon release from the mitochondria they become lethal to the cell. Release of cytochrome c from mitochondria into the cytoplasm initiates formation of the apoptosome, which causes cleavage and activation of caspase 9. This sets off a cascade of cleavages and activations of caspases, culminating in activation of executioner caspases 3, 6 and 7. These caspases cleave multiple protein targets including caspase-activated DNase (CAD/DFF40) resulting in DNA fragmentation, cellular shrinkage and blebbing into apoptotic bodies, and cell death. Also, when AIF becomes cleaved and released from damaged mitochondria, it translocates to the nucleus where it participates in a complex that promotes DNA fragmentation and chromatin condensation (Bano and Prehn, 2018).

Considering the SHetA2-causation of mitochondrial swelling and reduction of mitochondrial membrane potential in cancer cells, it is logical that SHetA2 treatment results in release of cell death factors from the mitochondria. In HNSCC, SHetA2 caused release of cytochrome c from the mitochondria leading to activation of caspase 3 and cell death through intrinsic apoptosis (Chun et al., 2003). Blockage of the mitochondrial membrane permeability transition pore (MPTP) with cyclosporin A reduced the SHetA2-induced release of cytochrome C and intrinsic apoptosis. An antioxidant also reduced SHetA2-induced apoptosis. This later finding is in contrast to the results of studies in ovarian cancer cells, which demonstrated that antioxidant reduction of ROS did not prevent SHetA2-induced apoptosis (Liu et al., 2007). In endometrial cells, SHetA2 also caused release of cytochrome c, as well as AIF, from the mitochondria leading to DNA damage and apoptotic cell death (Chandra et al., 2021). While the cellular recycling program of autophagy is induced by SHetA2, and can often protect against cell death, its induction in ovarian and endometrial cancer cells cannot protect against the ultimate mitochondrial release of cytochrome c and AIF (Benbrook and Long, 2012; Chandra et al., 2021). Currently it is not known if autophagy contributes to, or counteracts, SHetA2-induced cell death. It is feasible the upregulated autophagy counteracts cell death in the initial hours of SHetA2 treatment and then subsequently contributes to cell death by through excessive degradation/depletion of mitochondria and other factors required for survival.

The extrinsic apoptosis mechanism can also be induced by SHetA2. The extrinsic mechanism of apoptosis is initiated by plasma membrane death receptors and can be differentiated from intrinsic apoptosis by involvement of caspase 8 in extrinsic apoptosis and caspase 9 in intrinsic apoptosis. While SHetA2 caused only moderate and no cleavage of caspase eight in lung and ovarian cancer cells, respectively, it sensitized these cancer cells to caspase eight cleavage and apoptosis by death receptor ligands, tumor necrosis factor *α* (TNFα) and TNF-related apoptosis inducing ligand (TRAIL) (Lin et al., 2008a; Lin et al., 2008b; Moxley et al., 2009; Chengedza and Benbrook, 2010). One mechanism involved in this positive anti-cancer drug interaction is SHetA2 upregulation of CAAT/Enhancer Binding Protein Homologous Protein (CHOP), which leads to increased cell surface expression of the TNFα and TRAIL Death Receptors (DR4 and DR5) (Lin et al., 2008a; Chengedza and Benbrook, 2010). SHetA2 inhibition of Grp78 is a likely contributor to the mechanism of cell death because inhibition of GP78 was shown to enhance CHOP-mediated cell death in cancer cells (Luo et al., 2017). Another mechanism of SHetA2 regulation of extrinsic apoptosis involves SHetA2 repression of nuclear factor of kappa light polypeptide gene enhancer in B-cells (NF-κB). The NF-κB transcription factors are induced in the UPR response to ER stress and promote cell survival (van Antwerp et al., 1996). Although SHetA2 induces ER stress and UPR in cancer cells, it prevents the consequent upregulation of NF-κB by inhibition of the upstream kinases that induce NF-κB nuclear localization and transcriptional activities (Chengedza and Benbrook, 2010).

## Implication of Mortalin and Colleagues in SHetA2 Regulation of Cell Cycle and Angiogenesis

Mortalin and colleagues, Grp78 and hsc70, have numerous effects beyond the mitochondria that can be disrupted by SHetA2. The hsc70 chaperone supports cell cycle progression by binding to newly synthesized cyclin D1 and supporting the formation and function of the cyclin D1/cyclin dependent kinase 4 (CDK4) complex (Diehl et al., 2003). Additional hsc70 activities that could affect its regulation of cyclin D1/CDK4 complexes are the roles that it plays in chaperone mediated autophagy (CMA) and nucleocytoplasmic transport (Rai et al., 2021). In CMA, hsc70 binds proteins with an exposed KFERQ amino acid sequence caused by misfolding or disrupted complex formation, and brings the client proteins to the lysosome for degradation. In nucleocytoplasmic transport, hsc70 facilitates import of molecules into the nucleus by facilitating re-transport of importin receptor proteins back out to the cytoplasm for another round of importing after they have brought their cargo into the nucleus. The cellular localization of cyclin D1/CDK4 complexes is important because they need to be in the nucleus in order to exert its effect on the cell cycle. Mortalin is also known to bind cyclin D1 (Jirawatnotai et al., 2011), however the consequences of this binding have not been studied. Grp78 does not appear to directly regulate the cell cycle and has been shown to be dispensible for G1 arrest caused by induction of ER-stress with tunicamycin in hepatocellular carcinoma cells (Hsu et al., 2009).

The cyclin D1/CDK4 complex mediates G1 cell cycle progression by phosphorylating the retinoblastoma protein (Rb) and thereby relieving its inhibition of G1 cell cycle progression. SHetA2 treatment reduced cyclin D1 levels and induced G1 cell cycle arrest in both cancer and healthy cells, although the extents were greater in cancer over healthy cells (Liu et al., 2007; Liu et al., 2009; Masamha and Benbrook, 2009; Benbrook et al., 2017). The mechanism involved SHetA2 induction of cyclin D1 phosphorylation, ubiquitination and proteasomal degradation (Masamha and Benbrook, 2009), which could be a consequence of SHetA2 disruption of mortalin and hsc70 folding and support of this protein and its functional complex with CDK4 or CDK6. Overexpression of a non-degradable cyclin D1 mutant reduced SHetA2-induced G1 cell cycle arrest. The loss of cyclin D1 caused by SHetA2 led to release of p21 from cyclin D1/CDK4/6 complexes to bind and inhibit cyclin E2/CDK2 complexes resulting in decreased Rb phosphorylation. Decreased cyclin D1 and Rb phosphorylation, and induction of apoptosis were also observed to be caused by SHetA2 treatment of cervical cancer cell line xenograft tumors (Kennedy et al., 2020).

These molecular and cellular effects have been implicated in the mechanism by which SHetA2 inhibits tumor angiogenesis as observed through induction of G1 arrest in endothelial cells and inhibition of release of angiogenic growth factors from cancer cells (Myers et al., 2008). The ability of SHetA2 to induce G1 arrest in non-cancerous endothelial cells implies a direct effect of this compound on the ability of tumors to stimulate endothelial cells to form new blood vessels. SHetA2 interference with Grp78/client protein complexes is a likely upstream mediator of its inhibition of secretion of angiogenic factors from cancer cells. These cellular activities were associated with decreased expression of angiogenic factors, blockage and increased disorganization of blood vessels and development of ischemic necrosis observed in SHetA2-treated kidney cancer xenografts in contrast to higher levels of angiogenic factors, more-organized blood vessels and hemorrhagic necrosis in control tumors.

## Synergy of SHetA2 With Other Drugs

The multiple cellular effects of SHetA2 have been shown to enhance cancer cell sensitivities to a variety of other anti-cancer drugs that have complementary mechanisms of action (Table 2). In all instances, the combinations were shown to have greater efficacy without increasing toxicity. The synergy with TNFα and TRAIL described above was shown to be much-reduced in healthy cells (Moxley et al., 2009). Combination of SHetA2 with PRIMA-1^MET^, a drug that reactivates wild-type p53 apoptosis activity in mutant p53 proteins, also demonstrated complementary anti-cancer activity without toxicity in an orthotopic model of ovarian cancer maintenance therapy (Ramraj et al., 2019). In cell culture studies, SHetA2 and PRIMA-1^MET^ synergistically inhibited ovarian cancer cell line growth with greater effects in cell lines harboring missense mutant p53 and no significant drug interaction in healthy fallopian tube cell cultures. In the animal model, the two drugs acted additively in reducing the incidence of tumors without evidence of gross toxicity. PRIMA-1^MET^ (APR246) has been given orphan drug status for treatment of ovarian cancer in Europe, and thus offers a promising drug combination for translational development of SHetA2.

**TABLE 2:** Mechanisms of SHetA2 synergy with cancer drugs.

Synergistic drug categories	Mechanisms of action	Cancer types
Death Receptor Activators	SHetA2 upregulation of death receptors on the cell surface and repression of NF-κB activation and survival functions	Ovarian, Lung
p53 Reactivators	SHetA2 release of p53 from mortalin thereby allowing p53 translocation to the nucleus and mitochondria where the p53 reactivator allows the wild type p53 apoptosis-inducing activities	Ovarian
CDK4/6 Inhibitors	SHetA2 degradation of cyclin D1 (hypothesized to be caused by release of mortalin and hsc70 protection of cyclin D1 and the cyclin D1/CDK4/6 complex function) combined with inhibition of CDK4/6 activity in any remaining cyclin D1/CDK4/6 complexes	Cervical
Taxanes	Hypothesized to be caused by SHetA2 release of mortalin repression of p53’s control over centrosome reduplication thereby preventing continued DNA replication and cell survival in the presence of taxane-induced DNA damage	Endometrial

SHetA2 was also shown to synergize with the CDK4/6 inhibitor palbociclib in cervical cancer cell lines with additive effects in a xenograft model (Kennedy et al., 2020). The complementary mechanisms of these two drugs integrated at inhibition of cyclin D1/CDK4/6 phosphorylation of Rb through SHetA2-reduction of cyclin D1 and palbociclib-inhibition of the remaining cyclin D1/CDK4/6 complexes. Evaluation of the xenograft tumors confirmed complementary reduction of Rb phosphorylation, induction of apoptosis and inhibition of angiogenesis without evidence of toxicity in the single or drug combination treatment groups. Similar to the studies in kidney cancer xenografts, the blood vessels in the SHetA2-treated groups were enlarged and appeared to be blocked off causing adjacent areas of necrosis.

Complementation of SHetA2 with paclitaxel anti-cancer activity was predicted based on SHetA2 disruption of mortalin function at the centrosome and the ability of cancer cells to bypass taxane-induced cell death by re-duplicating centrosomes outside of the normal cell cycle process. Under normal cellular conditions, mortalin super-activates the monopolar spindle one kinase (Mps-1) to drive centrosome duplication and then p53 prevents more than one centrosome duplication per cell cycle (Kanai et al., 2007). In cancer cells, elevated mortalin interferes with the ability of p53 to repress centrosome re-duplication (Ma et al., 2006). This aberrant centrosome duplication allows DNA replication in the presence of DNA damage and thus continued proliferation and mutation of taxane-treated cancer cells. Studies of endometrial cancer cell lines documented that SHetA2 and paclitaxel acted synergistically to inhibit metabolic viability in cell culture and xenograft tumor growth without evidence of toxicity *in vivo* (Chandra et al., 2021).

## Cancer Chemoprevention Activities of SHetA2

Development of drugs for cancer prevention requires additional measures beyond the ability to inhibit or kill cancer cells. Because the target population for cancer chemoprevention drugs will be people who do not currently have cancer, only minimal-to-no side effects and ease of drug administration are essential. SHetA2 meets these requirements based on its NOAEL 50-fold above the predicted therapeutic dose and oral bioavailability. Development of a dietary formulation offers promise for using SHetA2 as a cancer prevention food additive (Benbrook et al., 2017). Development of SHetA2 inhaled and suppository formulations that provide increased drug levels to the target organs without systemic toxicity also offer less-invasive routes of administration for potential chemoprevention applications (Mahjabeen et al., 2018a; Mahjabeen et al., 2018b; Mahjabeen et al., 2018c; Ibrahim et al., 2018; Mahjabeen et al., 2020).

Initial evidence for SHetA2 chemoprevention activity was demonstrated using a 3-D organotypic culture model in which the carcinogen 7,12-dimethylbenz [a]anthracene (DMBA) was used to transform healthy endometrial epithelial cultures into the cancerous phenotype and SHetA2 reversed this phenotype back toward normal (Benbrook et al., 2008). The carcinogenesis and chemoprevention were observed by blinded pathology review of hematoxylin and eosin-stained fixed sections of the cultures, and were validated by independent karyometric analysis of the nuclear features and a soft agar clonogenic assay of cells released from the 3-D cultures. Bioinformatic analysis of mRNA changes in the cultures identified involvement of p53, TNFα and AP-1 signal transduction pathways in the mechanism of SHetA2 chemoprevention.

*In vivo* evidence of SHetA2 primary chemoprevention activity with oral bioavailability and without toxicity was acquired using the APC^min/+^ mouse model of colon and small intestinal tumorigenesis (Benbrook et al., 2013a). Oral treatment with SHetA2 at 30 or 60 mg/kg/day for 12 weeks reduced development of intestinal polyps by approximately 50% without evidence of toxicity. Heart function was specifically evaluated in this study out of concern that SHetA2 might damage mitochondria in healthy tissues, and the heart relies heavily on mitochondria for energy to drive its continuous beating. Magnetic resonance imaging of hearts in mice treated with either 60 mg/kg/day or control vehicle demonstrated no significant difference in stroke volume, cardiac output, ejection fraction or left ventricle wall thickness between the two groups demonstrating that SHetA2 did not cause cardiac toxicity. Comparison of tumors collected from the different treatment groups using immunohistochemistry and western blot analyses documented that SHetA2 reduced cyclin D1, proliferating cell nuclear antigen, Bcl-2, vascular endothelial growth factor, and cyclooxygenase 2, while increasing Bax and E-Cadherin.

## SHetA2 Clinical Trials

Currently, the investigational new drug application for the first in human clinical trials (IND 156700) has been allowed to proceed by the US FDA. This first clinical trial “Advanced or Recurrent Ovarian, Cervical, and Endometrial Cancer Treated with SHetA2 (Okgyn1)” (NCT04928508) is now open. It utilizes a capsule formulation based on Kolliphor HS15 (Ibrahim et al., 2019) and produced by the NCI PREVENT Cancer Preclinical Drug Development Program. The gynecologic cancer patient population was chosen based on these cancers having the most preclinical evidence of SHetA2 efficacy without toxicity. Because this is the first-in-human clinical trial of SHetA2, the advanced and recurrent population was chosen for ethical reasons. The trial design is a Phase 1 trial with the primary endpoints to determine dose-limiting toxicities and a dosage recommendation for Phase two trials (recommended phase two dose/RP2D). This RP2D could then be used in planned trials of SHetA2 drug combinations with death receptor activators, p53 reactivators, CDK4/6 inhibitors or paclitaxel. This RP2D could also be used in cancer chemoprevention trials.

Cancer prevention clinical trials of SHetA2 are also in development based on the preclinical efficacy of SHetA2 as a primary cancer chemoprevention agent in the APC^min/+^ model (Benbrook et al., 2013a) and secondary chemoprevention activity in the model of ovarian cancer maintenance therapy (Ramraj et al., 2019). The planned design of initial primary chemoprevention studies are window-of-opportunity biomarker trials to evaluate the effects of short-term SHetA2 treatment on normal or preneoplastic tissues. The patient population will be patients with pre-cancerous or other benign conditions scheduled for surgery as standard-of-care for non-cancerous reasons. Specimens from the treated group would be compared with specimens from a group of matched patients taking placebo or no drug. If the target tissue is accessible without surgery, such as in cervical tissue, then the patients can serve as their own controls via comparison of pre- and post-treatment biopsies. The pharmacodynamic biomarkers of SHetA2 to be evaluated in clinical trials include loss of cyclin D1 and Bcl-2 based on their down-regulation in association with SHetA2 cancer inhibition activities in multiple animal models described above. Additional biomarkers in development are based on modifications and cellular re-localization of mortalin and client proteins. In the cancer chemoprevention trials, surrogate biomarkers of cancer development also could be monitored.

Other HSP-targeted drugs have been developed and tested in clinical trials, however only one HSP90 inhibitor to date, AT13387, has been Food and Drug Administration (FDA)-approved for commercialization (Sanchez et al., 2020). MKT-077, a compound that inhibits mortalin was evaluated in clinical trials, however it was not advanced due to unacceptable off-target toxicity (Propper et al., 1999; Britten et al., 2000) apparently caused by nonspecific renal accumulation (Weisberg et al., 1996). Other HSP70-protein targeted drugs currently in cancer clinical trials are biologics being developed to elicit immune reactions for prevention or treatment (Rai et al., 2021). Currently, SHetA2 is the only small-molecule HSP70-inhibiting drug being studied in a clinical trial.

## Discussion

**FIGURE 3:**
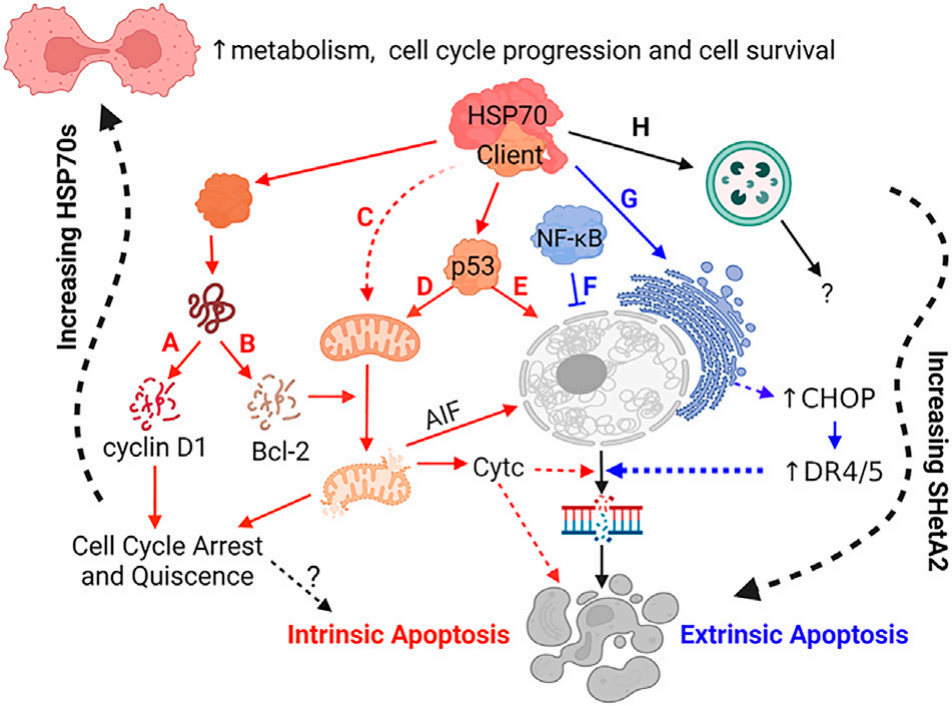
Mechanisms of SHetA2 Counteraction of Cancer Promotion by Mortalin, hsc70 and Grp78. SHetA2 counteracts the protection that mortalin, hsc70 and Grp78 give to cancer cells by disrupting their complexes with client proteins resulting in: **(A)** cyclin D1 phosphorylation, ubiquitination and proteasomal degradation (hypothesized to be caused by release of cyclin D1 from mortalin and hsc70) leading to reduction of Rb phosphorylation by the cyclin D1/CDK4/6 complex and G1 cell cycle arrest, **(B)** Bcl-2 degradation relieving Bcl-2 prevention of mitochondrial pore formation; **(C)** loss of mortalin functions in mitochondrial biogenesis, CA^2+^ homeostasis, membrane potential and oxidative phosphorylation leading to mitochondrial damage; **(D)** translocation of p53 from where it is bound by mortalin in the cytoplasm to the mitochondria where it promotes mitochondrial pore formation; **(E)** translocation of p53 from where it is bound by mortalin in the cytoplasm to the nucleus where it initiates transcription of apoptosis genes; **(A–E)** The events lead to mitochondrial release of cytochrome c (CytC) and AIF, which promote DNA and cell fragmentation leading to cell death through intrinsic apoptosis; **(F)** SHetA2 inhibition of kinase regulation of NF-κB and its nuclear translocation and survival functions, which allows induction of extrinsic apoptosis; **(G)** SHetA2 induction of ER stress and UPR (hypothesized to be caused by inhibition of Grp78) leading to induction of CHOP expression, which upregulates surface DR4 and DR5 expression and sensitivity to extrinsic apoptosis; **(F–G)** Mechanism sensitizing cells to extrinsic apoptosis; **(H)** Roles of SHetA2-induction of autophagy and plausible SHetA2 effects on CMA in the mechanisms of SHetA2-induced apoptosis are currently under investigation. Solid lines: direct effects, Dashed lines: indirect effects, Yellow lines: intrinsic apoptosis, Green lines: extrinsic apoptosis; Question Marks: Active areas of investigation; Squiggly line: gradual effects.

The translation of SHetA2 from concept through clinical trial supports the roles of its binding proteins, mortalin, hsc70 and Grp78 as anti-cancer drug development targets. The specific roles of these proteins as upstream SHetA2 targets and the involvement of their client protein alterations in the SHetA2 anti-cancer mechanism of action are now becoming apparent (Figure 3). The mechanisms of SHetA2 action defined to-date are guiding the development of clinical trials and will be important for interpreting efficacy and potential toxicities experienced by the patients.

Critical information needed in moving forward will be to determine the SHetA2 binding specificities to all of the HSP70 binding proteins and the consequent effects on their interactomes and cellular functions. There is potential that some of the SHetA2 HSP70/client protein disruptions are responsible for protecting healthy cells from SHetA2 toxicity or counteracting the cancer cell cytotoxicity of other SHetA2 HSP70/client protein interactions. Also, some of the various activities of individual SHetA2-targets could be counterproductive. For instance, SHetA2 disruption of hsc70/cyclin D1 complexes has the potential to inhibit CMA-degradation of cyclin D1 leading to increased cyclin D1 levels, while at the same time preventing hsc70 facilitation of cyclin D1/CDK4/6 complexes into its primary site of action in the nucleus. It does not appear however, that SHetA2 inhibits general nucleocytoplasmic transport, since SHetA2 treatment caused nuclear accumulation of p53 and NF-κB. Knowledge of the specific affinities of SHetA2 for the various HSP70 proteins and the timing of the consequent cellular activities is also needed to decipher the sequence of events leading to growth inhibition in healthy and cancer cells, and subsequent cell death in only cancer cells. Additional information needed includes profiling the SHetA2-targeted HSP70 interactomes of co-chaperones and client proteins in healthy and cancer cells under various non-stressed and stressed conditions. It will be important to know if SHetA2 disrupts all or only a subset of these complexes and the functions of the complexes and released client proteins.

Mechanistic understanding of SHetA2 action could be enhanced by integrating details of SHetA2 affinities and specificities for mortalin and other HPS70 proteins with knowledge of the timeline of SHetA2 alterations of HSP70 functions and down-stream released client proteins. This knowledge could then be integrated with the down-stream cellular consequences of SHetA2 treatment in healthy and cancer cells. A consequent understanding of the specific HSP70 and client protein disruptions and their time-lines in SHetA2-treated cancer and healthy cells could then be used to identify which HSP70 protein disruptions are responsible for, or counteract induction of, cancer cell death. Also, specific HSP70 disruptions that protect healthy cells from SHetA2 might be identified. Based on this integrated modeling, a strategy could be developed to refine the SHetA2 structure to have increased affinity for HSP70 interactions that are beneficial for killing cancer cells and protecting healthy cells, while avoiding any that may harm healthy cells or protect cancer cells. This effort could create a pipeline of Flex-Hets that could be tested for by-passing issues that may arise in the clinical trials and improve efficacies without adding toxicities to the compounds.
